# Developmental and Tissue Patterns of the Basal Expression of Chicken Avian *β*-Defensins

**DOI:** 10.1155/2020/2567861

**Published:** 2020-12-14

**Authors:** Wentao Lyu, Long Zhang, Yujie Gong, Xueting Wen, Yingping Xiao, Hua Yang

**Affiliations:** ^1^State Key Laboratory for Managing Biotic and Chemical Threats to the Quality and Safety of Agro-Products, Institute of Quality and Standard for Agro-Products, Zhejiang Academy of Agricultural Sciences, Hangzhou 310021, China; ^2^Institute of Ecology, Key Laboratory of Southwest China Wildlife Resources Conservation (Ministry of Education), China West Normal University, Nanchong 637009, China

## Abstract

Defensins are a class of antimicrobial peptides in vertebrates that function as the first line of innate immunity with potent antimicrobial and immunomodulatory activities. Fourteen defensins, namely, avian *β*-defensin 1 to 14 (*AvBD1*-14), have been identified in chickens. Before characterizing the role of *AvBD*s in innate immunity during the early development of chickens, we collected tissue segments from the liver, spleen, and gastrointestinal (GI) tract including the esophagus, crop, proventriculus, gizzard, duodenum, jejunum, ileum, cecum, and colon from broilers at days 1, 3, 7, 14, and 28. After RNA isolation and reverse transcription, we determined the expression levels of the 14 *AvBD* genes in these tissues during the first 28 days after hatching by real-time PCR. The results suggested the *AvBD*s were widely expressed in the chicken liver, spleen, and gastrointestinal (GI) tract. Interestingly, we did not detect *AvBD11* expressed in the GI tract, even in the liver and spleen. Additionally, *AvBD*s were differentially expressed in the chicken GI tract. *AvBD5* and *AvBD14* were expressed most abundantly in the proximal GI tract, especially the esophagus and crop. Moreover, *AvBD5*, *AvBD7*, *AvBD9*, and *AvBD14* were expressed in an inverted-V pattern with the peak being the observed expression at days 3, 7, or 14 in the chicken spleen, esophagus, duodenum, and cecum. Other *AvBD*s presented biphasic or inverted-V expression patterns in different tissues. The expression levels of all detected *AvBD*s were strengthened after hatching rather than decreasing steadily. Therefore, *AvBD*s were found to be expressed widely in the chicken liver, spleen, and GI tract and their expression levels were primarily up regulated during the early development of chicken, implying the potential essential roles of *AvBD*s in early innate defense and infection resistance of chickens.

## 1. Introduction

Due to the overuse of antibiotics in farm animals, it would raise a high risk of transferring antibiotic resistance to humans, which may threaten public health. Therefore, the development of novel antibiotics is an urgent need and is attracting increasing attention. Enhancing the synthesis of host defense peptides (HDPs) has emerged as a host-directed antibiotic alternative therapy showing less likelihood of triggering antibiotic resistance [1, 2]. As an essential part of innate immunity, HDPs, also known as antimicrobial peptides, are small peptides consisting of less than 100 amino acid residues. These peptides include various groups of small peptides defending against environmental pathogens. Besides, HDPs are widely distributed in almost all species of life [3–5].

For vertebrates, HDPs consist of two major families: cathelicidins and defensins. HDPs are mainly synthesized and secreted by phagocytic cells and the cells of the epithelial surfaces such as the skin and the respiratory, gastrointestinal (GI), urogenital, and reproductive tracts [3, 6], suggesting that HDPs act as the first line of defense against microbes. Chickens express 4 cathelicidins (CATH) and 14 avian *β*-defensins (*AvBD*s) with no *α*- and *θ*-defensins, namely, CATH1-3 (also known as fowlicidin 1-3), CATH-B1, and *AvBD1-14* [7]. Accumulating evidence indicates that *AvBD*s have wide-spectrum microbiostatic activities against gram-negative and gram-positive bacteria, fungi, and viruses [8, 9]. For instance, purified recombinant *AvBD6* protein could inhibit the growth of *Escherichia coli*, *Campylobacter jejuni*, *Clostridium perfringens*, *Staphylococcus aureus*, *Saccharomyces cerevisiae*, and *Candida albicans* [10]. Recombinant *AvBD2* was able to lower the cytotoxicity of the Newcastle disease virus [8]. Exhibiting potent antimicrobial activities, HDPs are considered a potential antibiotic alternative strategy.

The GI tract acts as the digestive tract to extract nutrients from food for chickens and includes the esophagus, crop, proventriculus, gizzard, duodenum, jejunum, ileum, cecum, and colon [11]. *AvBD*s synthesized by epithelial cells lining the GI tract are essential weapons by which chickens could combat the millions of microbes trying to invade through different pathways [9, 12, 13]. In the GI tract, *AvBD9* is one of the most widely spread *AvBDs* and is found in the esophagus, crop, proventriculus, gizzard, duodenum, ileum, and colon [10, 14, 15]. However, few studies have been conducted on the developmental and tissue patterns of *AvBD* basal expression in chickens at an early age.

In this study, we examined the developmental and tissue expression patterns of *AvBD*s in broiler chickens during the first 28 days after hatching. We noticed that each *AvBD* had tissue specificity and that *AvBD*s would give a higher expression during the first two weeks than on day 1 after hatching, indicating that *AvBD*s are significant for chickens to fight against pathogens during early development.

## 2. Materials and Methods

### 2.1. Animals and Sampling

The experiments were approved by the Animal Care and Use Committee of Zhejiang Academy of Agricultural Sciences. A total of 90 newly hatched male Ross 308 broiler chicks were raised in a controlled environment at a temperature of 34-35°C in the first week followed by a reduction of 3°C weekly to a final temperature of 26°C. The broilers received no antibiotics throughout the experimental period. Tissue samples (*n* = 9) were collected on days 1, 3, 7, 14, and 28 from the esophagus, crop, gizzard, proventriculus, duodenum, jejunum, ileum, cecum, colon, liver, and spleen of 3 chickens. All the tissue samples were frozen in liquid nitrogen immediately and stored at -80°C until RNA isolation.

### 2.2. RNA Extraction and Quantification

Total RNA was isolated from tissues using a TRIzol® Plus RNA Purification Kit (Invitrogen, Grand Island, NY, USA) according to the manufacturer's instructions. The concentrations and qualities of RNA were measured by a NanoDrop One Spectrophotometer (Beckman Coulter, Brea, CA, USA).

### 2.3. Reverse Transcription and Quantitative PCR

The first-strand cDNA was synthesized from 300 ng of RNA using SuperScript™ III First-Strand Synthesis SuperMix (Invitrogen) and was then diluted into 40 *μ*l with RNase-free water. Real-time quantitative PCR was conducted on a CFX384 Touch™ Real-Time PCR Detection System (Bio-Rad, Hercules, CA, USA) in a 20 *μ*l system including 1 *μ*l of diluted cDNA, 0.5 *μ*l of both specific forward and reverse primers (Table 1), 10 *μ*l of Power SYBR® Green PCR Master Mix (Applied Biosystems, Carlsbad, CA, USA), and 8 *μ*l of water. The primers were designed for the specific 15 genes by Primer Premier 6.0 (Premier Biosoft, Palo Alto, CA, USA) and Beacon designer 7.8 (Premier Biosoft). The PCR program was 95 for 1 min, 40 cycles of 95 for 15 s, and 63 for 25 s. To confirm the specificity of the PCR, melting curves were detected. The fold change of gene expression was calculated through the comparative *ΔΔ*Ct method, setting the glyceraldehyde-3-phosphate dehydrogenase (GAPDH) gene as the housekeeping gene for data normalization [16].

### 2.4. Statistical and Correlation Analyses

The Pearson correlation coefficient was calculated as previously described using SPSS Statistics 23.0.0.0 (IBM, Armonk, NY, USA) [17]. OriginPro 2018 (OriginLab Corporation, Northampton, MA, USA) was used for data visualization and analysis with one-way ANOVA. The data are expressed as means ± standard error of means (SEM). *P* value < 0.05 was considered to indicate significant differences.

## 3. Results

### 3.1. Abundance of AvBDs in Chickens

**Figure 1:**
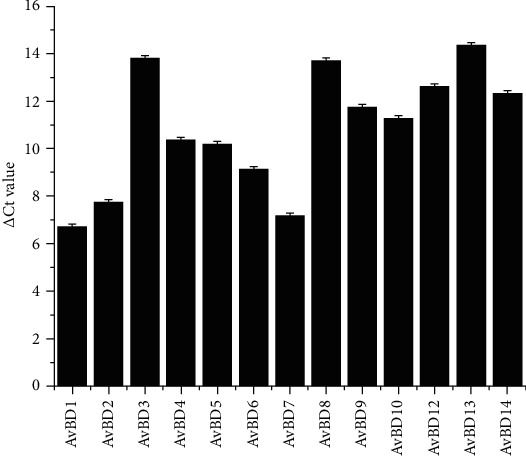
Average ΔCt values of chicken *AvBD*s in the GI tract. Tissues of the GI tract were obtained from chickens at indicated ages. RNA isolation and real-time PCR analysis were performed to evaluate the expression levels of all detected chicken *AvBD*s. *Δ*Ct values of chicken *AvBD*s were calculated relative to those of the colon on day 28 using *GAPDH* as the reference gene and are expressed as the mean ± standard error of the mean of nine chickens.

To evaluate the expression abundance of *AvBD*s in the chicken GI tract, we collected various tissues along the chicken GI tract from eighteen 14-day-old Ross broiler chickens and subjected the tissues to total RNA isolation and RT-qPCR. The average *Δ*Ct values were calculated to indicate the *AvBD1-14* expression abundances, with normalization to GAPDH expression. As expected, all *AvBD*s except *AvBD11* were widely expressed in the chicken esophagus, crop, proventriculus, gizzard, duodenum, jejunum, ileum, cecum, and colon with different levels of abundance (Figure 1). The small SEM implies that each *AvBD* gave a similar expression level in each segment of the chicken GI tract. As shown in Figure 1, among all the detected *AvBD*s, *AvBD1* was the most abundantly expressed (mean of ΔCt = 6.70) while *AvBD13* was the least abundant one (mean of ΔCt = 14.37) in the chicken GI tract. Additionally, *AvBD11* was expressed at the lowest level in the chicken GI tract, as its expression was not detected in the present study.

### 3.2. Differential Tissue Pattern of Chicken AvBDs

**Figure 2:**
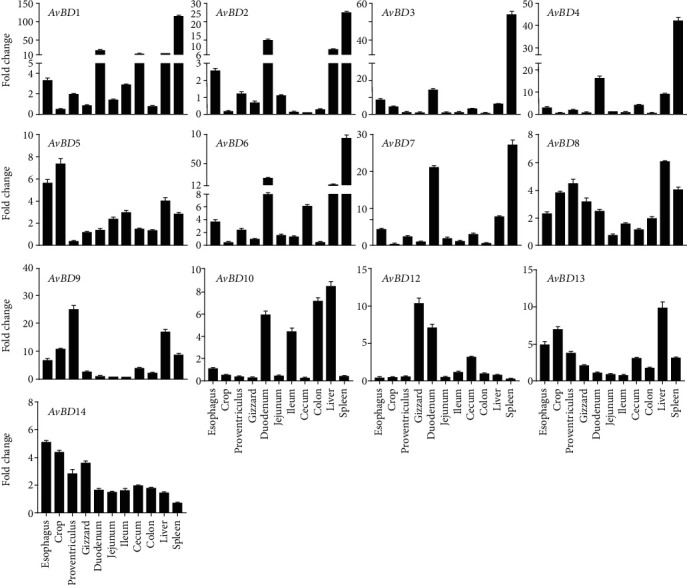
Tissue patterns of chicken *AvBD*s along the GI tract, liver, and spleen. Tissue segments of the esophagus, crop, proventriculus, gizzard, duodenum, ileum, cecum, colon, liver, and spleen were obtained from chickens at 7-day-old. After RNA isolation, RT-qPCR analysis was performed to evaluate the mRNA expression levels of all chicken *AvBD*s. Fold changes of chicken *AvBD* expression were calculated relative to the expression level of the colon on day 28 using *GAPDH* as the reference gene and are expressed as the mean ± standard error of the mean of nine chickens.

To study the tissue expression patterns of *AvBD*s along the chicken GI tract, we collected tissue samples from the liver, spleen, and the GI tract, including the esophagus, crop, proventriculus, gizzard, duodenum, jejunum, cecum, and colon, from 7-day-old Ross chickens because most of the *AvBD* expression was expressed at relatively high levels in 7-day-old chickens.The basal mRNA expression levels of *AvBD1-14* were examined by RT-qPCR after RNA extraction and reverse transcription. The average *Δ*Ct values of all the detected *AvBD*s in the chicken GI tract ranged from 6.70 to 14.37, and the average was 10.87 (Figure 1). Accordingly, among all the detected defensins, all but *AvBD11* were widely expressed with different patterns along the chicken GI tract and in the liver and spleen (Figure 2). As shown in Figure 2, *AvBDs 1-4*, *6*, and *7* shared a common expression pattern; i.e., the chicken spleen exhibited the highest expression levels of those *β*-defensins compared to other tissues at 7-day-old, whereas the duodenum gave the highest expression level in *AvBDs 1-4*, *6*, and *7*. Additionally, the difference between the highest and lowest relative mRNA expression levels of *AvBDs 1-4*, *6*, and *7* was more than 40-fold and was as high as 178-fold (Figure 2). Another differentially expressed *β*-defensin was *AvBD9*; the difference between the highest and lowest expression was as much as 25-fold. However, *AvBD9* was expressed at a relatively higher level in the proventriculus than in other tissues, even compared to the levels in the liver and spleen. Besides, the liver showed a higher *AvBD9* mRNA expression level than the spleen. For the other *AvBD*s (*AvBD5*, *AvBD8*, *AvBD10*, *AvBD12*, and *AvBD13*), the mRNA expression levels in different tissues were not very different (Figure 2). The relative fold changes of these *AvBD*s were no more than 15-fold. *AvBD11* was not detected in the chicken GI tract because the absolute Ct value of *AvBD11* was above 35.

Among chicken digestive tissues, it was also observed that the duodenum yielded the highest expression of *AvBDs 1*-*4*, *6*, and *7*, while the crop and proventriculus gave the highest expression levels of *AvBD5*, *AvBD13*, *AvBD8*, and *AvBD9*, respectively (Figure 2). Furthermore, *AvBD12* and *AvBD14* were highly expressed in the gizzard and esophagus, respectively. Esophagus and crop shared the same patterns of the *AvBD* expression (Supplemental Figure (see available here)). Particularly, *AvBD10* was expressed most in the colon, duodenum, and cecum with up to 7.14-fold change while *AvBD9* was the most abundantly expressed in the proventriculus with fold change up to 24.82 (Supplemental Figure (see available here)).

### 3.3. Developmental Expression Patterns of Chicken AvBDs

**Figure 3:**
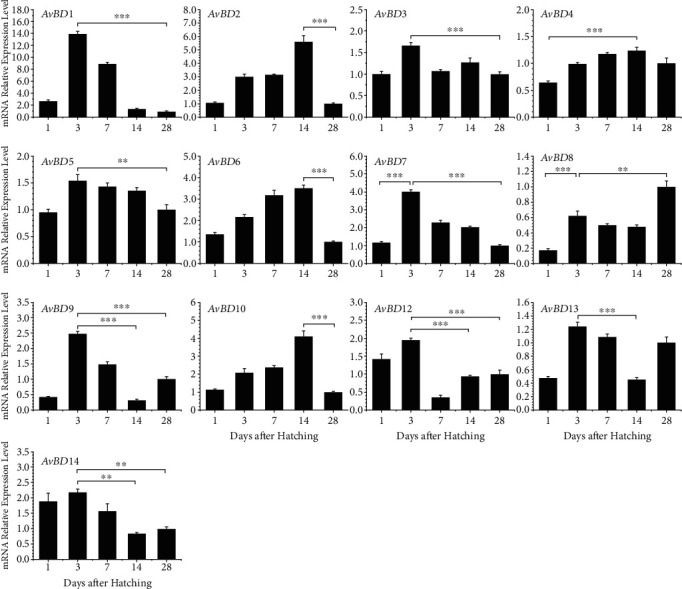
Developmental expression of chicken *β*-defensins in the spleen. The spleens were harvested from broilers at 1, 3, 7, 14, and 28 days old and were subjected to RT-qPCR analysis after RNA isolation and reverse transcription. mRNA expression levels in *β*-defensins of indicated ages were calculated as fold changes relative to the expression level on day 28 using *GAPDH* as the house-keeping gene. Each bar represents the mean ± standard error of the mean of three chickens. The difference was considered significant using one-way ANOVA followed by Tukey's test. ^∗^*P* < 0.05; ^∗∗^*P* < 0.01; ^∗∗∗^*P* < 0.001.

To investigate the dynamic expression patterns of fourteen *AvBD*s at an early age, we collected esophagus, crop, proventriculus, gizzard, duodenum, jejunum, ileum, cecum, and colon segments from Ross chickens at 1, 3, 7, 14, and 28 days after hatching. *AvBD* gene expression levels were determined by RT-qPCR after RNA isolation and reverse transcription. Overall, clear differential expression patterns with all detected chicken *AvBD*s were observed. In the spleen, the expression levels of most *β*-defensins, namely, *AvBD1*, *AvBD3*, *AvBD5*, *AvBD7*, *AvBD9*, *AvBD12*, *AvBD13*, and *AvBD14*, peaked 3 days after hatching and then gradually decreased, with the lowest expression being observed on day 14 or 28 relative to day 4 (Figure 3). The mRNA expression levels of *AvBD2*, *AvBD4*, *AvBD6*, and *AvBD10* showed similar patterns, with the expression peak on day 14 (Figure 3). Interestingly, the highest expression of *AvBD8* was observed on day 28, where the difference was significant (*P* < 0.001). Significant downregulations (*P* < 0.01) in the expression levels of *AvBD1*, *AvBD3*, *AvBD5*, *AvBD7*, *AvBD9*, *AvBD12*, and *AvBD14* were observed in the spleen on day 28 relative to day 3 while the expression levels of *AvBD2*, *AvBD6*, and *AvBD10* significantly attenuated on day 28 relative to day 14 (*P* < 0.001). The mRNA expression level of *AvBD1* was significantly decreased by nearly 14-fold between day 3 and day 28 (Figure 3).

**Figure 4:**
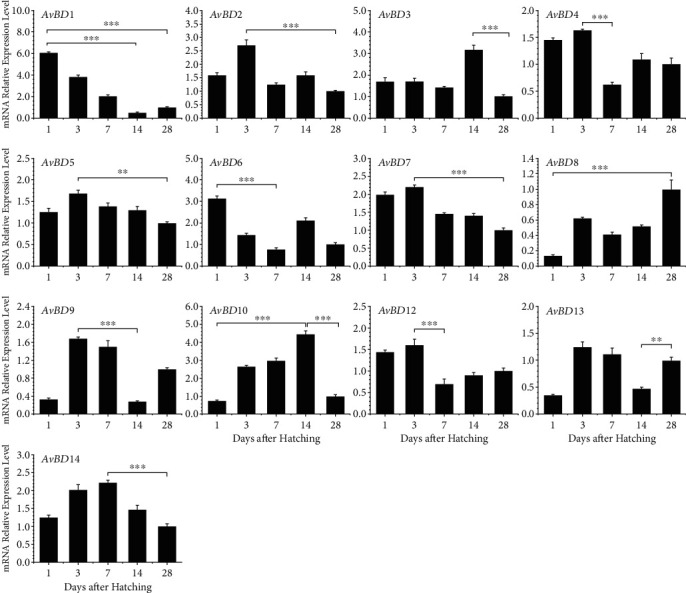
Developmental expression of chicken *β*-defensins in the esophagus. Esophagus segments were harvested from broilers at 1, 3, 7, 14, and 28 days of age and were subjected to RT-qPCR analysis after RNA isolation and reverse transcription. The mRNA expression levels of *β*-defensins at the indicated ages were calculated as fold changes relative to the expression level on day 28 using *GAPDH* as the housekeeping gene. Each bar represents the mean ± standard error of the mean of three chickens. The difference was considered significant using one-way ANOVA followed by Tukey's test. ^∗^*P* < 0.05; ^∗∗^*P* < 0.01; ^∗∗∗^*P* < 0.001.

**Figure 5:**
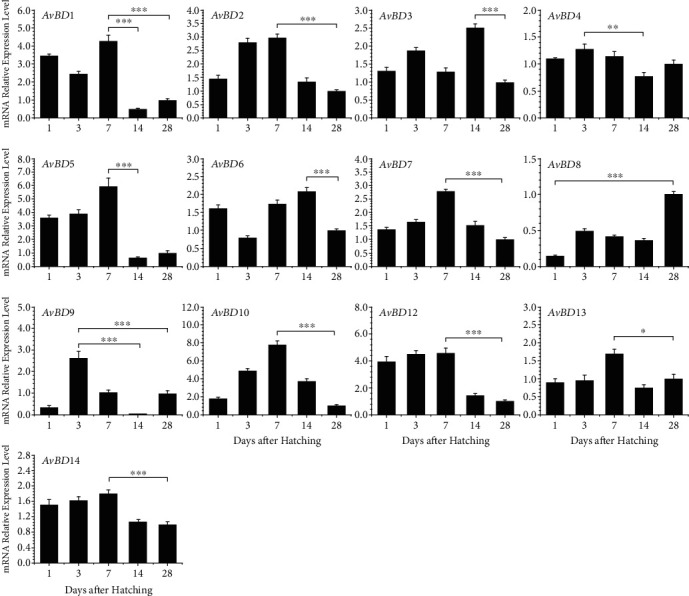
Developmental expression of chicken *β*-defensins in the duodenum. The duodenums were harvested from broilers at 1, 3, 7, 14, and 28 days of age and were subjected to RT-qPCR analysis after RNA isolation and reverse transcription. The mRNA expression levels of *β*-defensins at the indicated ages were calculated as fold changes relative to the expression level on day 28 using *GAPDH* as the housekeeping gene. Each bar represents the mean ± standard error of the mean of three chickens. The difference was considered significant using one-way ANOVA followed by Tukey's test. ^∗^*P* < 0.05; ^∗∗^*P* < 0.01; ^∗∗∗^*P* < 0.001.

**Figure 6:**
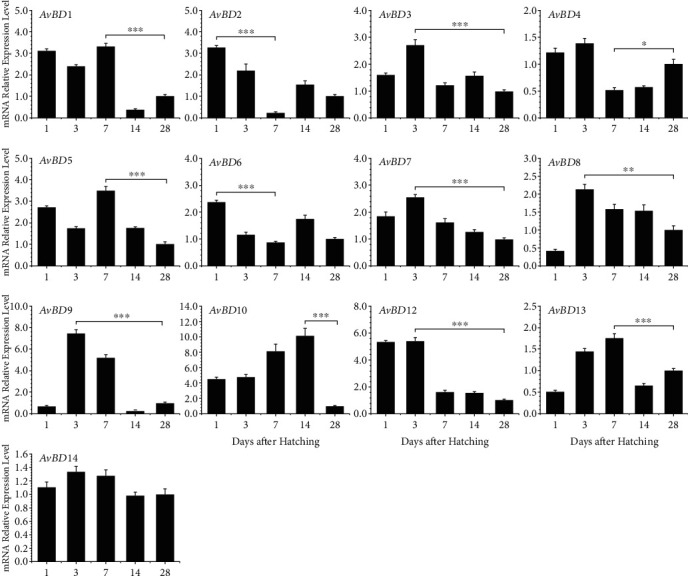
Developmental expression of chicken *β*-defensins in the cecum. The ceca were harvested from broilers at 1, 3, 7, 14, and 28 days of age and were subjected to RT-qPCR analysis after RNA isolation and reverse transcription. The mRNA expression levels of *β*-defensins at the indicated ages were calculated as fold changes relative to the expression level on day 28 using *GAPDH* as the housekeeping gene. Each bar represents the mean ± standard error of the mean of three chickens. The difference was considered significant using one-way ANOVA followed by Tukey's test. ^∗^*P* < 0.05; ^∗∗^*P* < 0.01; ^∗∗∗^*P* < 0.001.

In the digestive tract, the *AvBD* expression patterns were more complex. Biphasic expression patterns showed up in the esophagus (Figure 4), duodenum (Figure 5), cecum (Figure 6), and other digestive tissues (Supplemental Figure (see available here)). In the esophagus, *AvBD1*, *AvBD4*, *AvBD6*, *AvBD8*, *AvBD9*, *AvBD12*, and *AvBD13* gave a biphasic expression pattern, where these genes were abundantly expressed on day 1 or day 3 but eventually reduced to the lowest expression on day 7 or day 14 (Figure 4). Additionally, *AvBD2*, *AvBD3*, *AvBD5*, *AvBD7*, *AvBD10*, and *AvBD14* showed peak expression levels at different days with the lowest expression being observed on day 28. The duodenum and cecum showed similar expression patterns (Figures 5 and 6). Interestingly, no significant difference in *AvBD14* expression level was observed in the cecum at different ages (Figure 6). For *AvBD8*, the spleen, esophagus, and duodenum shared the same expression pattern, where *AvBD8* expression was increased to the first peak on day 3 but slightly reduced from day 7 to day 14, followed by the highest expression on day 28.

### 3.4. Correlations among Chicken AvBD Expression in the Chicken Digestive Tract

To reveal the correlations among *AvBD*s expression in the chicken GI tract, correlations were determined using the Pearson correlation coefficient. The expression levels of half of the *AvBD*s were significantly correlated with chicken age, suggestive of the age specificity of *AvBD* expression. As stated in Table 2, the correlation coefficients of *AvBD2* vs. *AvBD4*, *AvBD4* vs. *AvBD6*, *AvBD4* vs. *AvBD7*, and *AvBD6* vs. *AvBD7* were all more than 0.8 with high significance (*P* < 0.01), showing a strong positive correlation between these *AvBD* expressions.

## 4. Discussion

The digestive system in chickens consists of the esophagus, crop, proventriculus, gizzard, small intestine (duodenum, jejunum, and ileum), cecum, colon, and cloaca [18]. The broad *AvBD* distribution along the digestive tract makes these organs function as barriers in preventing pathogen invasion into the GI tract [7]. The present study showed *AvBD*s were widely expressed in the chicken GI tract except for *AvBD11*, which coincides with the previous study [9, 15]. Although the expression levels of *AvBD*s and other host defense peptides gave a gradual increase followed by a dramatic decrease in the sterile environment during the embryonic development of chickens [19, 20], we found the *AvBD* expression levels were further strengthened during the first 28 days after hatching. Because chicks are constantly exposed to different pathogens in the ambient environment with inadequate protection of circulating maternal antibodies [21], these results might not be surprising. With the presence and rapid development of innate immunity in chicks, the adaptive immunity gradually matures to prevent various pathogen infections in newly hatched chickens. The heightened expression of *AvBD*s could contribute essential protection against environmental microbial invasion in the early stage of chickens with potent antimicrobial activities. For detected *AvBD*s, different tissues showed their preferences for *AvBD* expression. For instance, *AvBD1-4* and *AvBD6-7* were strongly expressed in the duodenum, and *AvBD5* and *AvBD13*-14 were strongly expressed in the proximal GI tract including the esophagus and crop (Figure 2 and Supplemental Figure (see available here)). Additionally, the high expression levels of *AvBD8-9* and *AvBD12* were observed in the proventriculus and gizzard, respectively. Interestingly, the distal GI tract was relatively rich in only *AvBD10* and *AvBD12*. *AvBD*s function not only as antimicrobials but also as sensors of the host-microbiome balance along the digestive tract [22]. Our results provide evidence that the cecum might be tolerant to microbes for further feed digestion. We also found similar patterns of *AvBD* expression in the esophagus and crop, possibly because the only mucosal glands in the crop are located in the junction of the esophagus and crop [11].

The majority of defensins are broadly expressed in a wide range of cells in the GI tract [22, 23]. Typically, some *α*-defensins are uniquely synthesized in Paneth cells in the crypts in the small intestine [24–26]. However, the chicken genome has no *α*-defensin gene, and a recent study indicated the presence of Paneth cells in the chicken small intestine [27]. These observations make it particularly interesting to investigate the expression patterns of *β*-defensins in the chicken GI tract. Our results showed that all but *AvBD11* were widely expressed in the tissues of the GI tract. The absence of *AvBD11* mRNA in the GI tract is probably due to its unique gene structure, which contains two tandem copies of the six-cysteine motif in the mature peptide [28]. However, the expression of *AvBD11* was detected in the crop and intestinal mucosal layer of Cobb and Ross broiler chickens and its expression would increase in the intestinal mucosal layer when the Cobb broilers were challenged with *Eimeria maxima* [29]. The chicken species used in this study and our focus on the basal expression level of *AvBD*s might be another reason why we did not detect *AvBD11* expression. Simultaneously, we noticed that multiple significant correlations were present among some genes. Coupled with the locations and phylogenetic relationships of chicken defensins, we also found that many of the genes expressed with high correlations are located close or even adjacent to each other and are clustered in the same gene clade. For instance, all of the correlations among *AvBD1-4* were significant with correlation coefficient values higher than 0.5 (Table 2). Regarding *AvBD6*, which is a duplicate from *AvBD7* in Galliformes with a short chromosome distance [15], it also presented a strong positive correlation with *AvBD7*. Interestingly, significant correlations with high correlation coefficients are also observed in some genes with long chromosome distances and low similarities. For example, the correlation between *AvBD9* and *AvBD13* is highly significant with a correlation coefficient of 0.88, while the two genes not only show less similarity at the amino acid level but are also located in different *AvBD* gene blocks.

As represented by *AvBD1*, the expression levels of multiple *AvBD*s tended to be decreased from day 1 to day 28 in both the GI tract and the liver and spleen. The decreased expression levels of *AvBD*s might be associated with the maturation of adaptive immunity. In contrast, the mRNA abundance of *AvBD8* pervasively increased along with age, which is notably different from the other *AvBD*s. On the account of the narrow antibacterial range of *AvBD8* in chickens, the primary biological function of this gene is unlikely to be antimicrobial [30]. In fact, defensins represent a posse of pleiotropic molecules that exert many other effects in the immune system beyond host defense. Therefore, the gradual increasing of the *AvBD8* expression in these tissues may relate to other functions or may act synergistically with other defensins to exert defense functions. Different from the liver and spleen, the GI tract continuously interacts with microbes. It has been known that defensins are able to balance among bacterial populations and to control homeostasis in the GI tract [31]. Chicken CHCC-OU2 cells challenged *in vitro* by commensal gut bacteria lead to significant expression changes in some *AvBD*s [32]. Therefore, some biphasic expression patterns, such as the abrupt elevation in the duodenum at day 7, may be the result of microbe colonization. Meanwhile, some microbial productions can also stimulate the expression of chicken *AvBD*s. Butyrate, a kind of short-chain fatty acid produced by *Clostridium butyricum*, is capable of inducing a group of *AvBD*s in multiple cell types [33]. This finding may indirectly regulate the expression of some *AvBD* genes in the GI tract. The expression levels of *AvBD*s in the liver and spleen are abundant because they are important immune organs with a large amount of immune cells. The different expression patterns of *AvBD*s in these tissues may also be associated with their biological functions.

## 5. Conclusions

Taken together, the expressions of *AvBD*s in the GI tract are gene-, tissue- and age-specific. Influenced by the living environment, health condition, and genetic background, conflicting expression patterns are observed for *AvBD*s in different studies [9, 28, 32]. Continued studies focus on the identification of the cell types that synthesize specific *AvBD*s and investigations of *AvBD* mRNA abundance by using germ-free chickens, which would improve our understanding of the expression patterns of this gene family.

## Figures and Tables

**Table 1:** Primer sequences of chicken AvBDs for real-time PCR.

Gene	Accession No.	Primer sequences (5′→3′)
*Gallus AvBD1*	NM_204993.1	F: CACCCTGGCTTCTCGCTTCTG
		R: GTGGGATGTCTCCAACTTCTACTG
*Gallus AvBD2*	DQ677633.1	F: CACTCCAGGTTTCTCCAGGGTT
		R: CGAAGCAGCTTCCGACTTTGAT
*Gallus AvBD3*	NM_204650.2	F: AGGATTCTGTCGTGTTGGGAGC
		R: TTCCAGGAGCGAGAAGCCAC
*Gallus AvBD4*	NM_001001610.2	F: GGGCTATGCCGTCCCAAGT
		R: GGTTCCCCAAATCCAACAATGC
*Gallus AvBD5*	NM_001001608.2	F: GAGCCGATGGTATTCCTGATGG
		R: GTGGTGATTGTTGCCTCTGGTG
*Gallus AvBD6*	NM_001001193.1	F: GTTGGATCATGTGGCAGTGGAC
		R: CAGCAGGTTGGATGGAGTTAGAG
*Gallus AvBD7*	NM_001001194.1	F: CAATGGAATAGGCTCTTGCTGTG
		R: GTGCCAGATAGAATGGAGTTGGAG
*Gallus AvBD8*	NM_001001781.1	F: GGATCACTGCTTCCACCTCCATAC
		R: GGTCTGAGGTCCTGGCGAACA
*Gallus AvBD9*	NM_001001611.2	F: CTGCCTTATGACATCACTGGATCTTT
		R: TCGTGCTCCCAGGACTCTTC
*Gallus AvBD10*	NM_001001609.2	F: TGGGGCACGCAGTCCACAAC
		R: CAATCAGCTCCTCAAGGCAGTG
*Gallus AvBD11*	NM_001001779.1	F: GCAGAAAGCCACAGAAGTGC
		R: CGTCGCCTCTAACGAATTGCA
*Gallus AvBD12*	NM_001001607.2	F: CACCAACTCCCACCAAGACCT
		R: GCAAGTGAATCCACAGCCAATGAGA
*Gallus AvBD13*	NM_001001780.1	F: GCTCTTTGCCATCGTTGTCATTCTC
		R: CTCCATGTGGAAGCAGAGCCT
*Gallus AvBD14*	AM402954.1	F: GGCATATTCCTCCTGTTTCTTGTTC
		R: CTTGCCCTTCATCTTCCGACA
*Gallus GAPDH*	NM_204305.1	F: CAGAACATCATCCCAGCGTCCA
		R: ACGGCAGGTCAGGTCAACAA

**Table 2:** Pearson correlation among GI tract tissues, age of chickens, and AvBDs.

	Age	*AvBD1*	*AvBD2*	*AvBD3*	*AvBD4*	*AvBD5*	*AvBD6*	*AvBD7*	*AvBD8*	*AvBD9*	*AvBD10*	*AvBD12*	*AvBD13*	*AvBD14*
Tissue	0.000	0.096	-0.180^∗^	-0.242^∗∗^	0.017	-0.626^∗∗^	0.006	-0.082	-0.488^∗∗^	-0.322^∗∗^	0.380^∗∗^	0.055	-0.564^∗∗^	-0.626^∗∗^
Age		-0.445^∗∗^	-0.202^∗^	-0.037	-0.109	-0.139	-0.166	-0.187^∗^	0.354^∗∗^	-0.103	-0.176^∗^	-0.292^∗∗^	0.034	-0.307^∗∗^
*AvBD1*			0.672^∗∗^	0.420^∗∗^	0.743^∗∗^	-0.080	0.679^∗∗^	0.763^∗∗^	-0.289^∗∗^	-0.132	0.189^∗^	0.385^∗∗^	-0.169^∗^	-0.049
*AvBD2*				0.748^∗∗^	0.819^∗∗^	-0.010	0.668^∗∗^	0.924^∗∗^	-0.018	-0.073	0.353^∗∗^	0.204^∗^	-0.108	0.019
*AvBD3*					0.755^∗∗^	0.126	0.779^∗∗^	0.773^∗∗^	-0.053	-0.160	0.221^∗∗^	0.090	-0.054	0.085
*AvBD4*						-0.162	0.807^∗∗^	0.870^∗∗^	-0.099	-0.134	0.179^∗^	0.338^∗∗^	-0.169	-0.095
*AvBD5*							-0.138	-0.064	0.172^∗^	0.182^∗^	-0.102	-0.300^∗∗^	0.637^∗∗^	0.691^∗∗^
*AvBD6*								0.817^∗∗^	-0.236^∗∗^	-0.250^∗∗^	0.144	0.239^∗∗^	-0.266^∗∗^	-0.162
*AvBD7*									-0.094	-0.105	0.362^∗∗^	0.262^∗∗^	-0.130	-0.009
*AvBD8*										0.520^∗∗^	-0.176^∗^	-0.119	0.568^∗∗^	0.244^∗∗^
*AvBD9*											-0.210^∗^	-0.181^∗^	0.698^∗∗^	0.463^∗∗^
*AvBD10*												-0.047	-0.274^∗∗^	-0.136
*AvBD12*													-0.236^∗∗^	0.074
*AvBD13*														0.702^∗∗^

^∗^ *P* < 0.05; ^∗∗^*P* < 0.01; ^∗∗∗^*P* < 0.001.

## Data Availability

The data used to support the findings of this study are included within the article.

## Supplementary Materials

Supplementary Materials Supplementary Figure: basal expression of thirteen chicken *AvBD*s in the GI tract, liver, and spleen. Tissue segments were collected from 7-day-old chickens. After RNA extraction and reverse transcription, *AvBD* expression levels were determined by real-time PCR using gene-specific primers. The expression levels of all *AvBD*s were calculated relative to that of the colon using *GAPDH* as the reference gene and expressed as the mean of nine chickens for each time point.

## References

[1] Lyu W., Curtis A. R., Sunkara L. T., Zhang G. (2015). Transcriptional regulation of antimicrobial host defense peptides. *Current Protein & Peptide Science*.

[2] Lyu W., Deng Z., Sunkara L. T. (2018). High throughput screening for natural host defense peptide-inducing compounds as novel alternatives to antibiotics. *Frontiers in Cellular and Infection Microbiology*.

[3] Zasloff M. (2002). Antimicrobial peptides of multicellular organisms. *Nature*.

[4] Zhang L., Chen D., Yu L., Wei Y., Li J., Zhou C. (2019). Genome-wide analysis of the ovodefensin gene family: monophyletic origin, independent gene duplication and presence of different selection patterns. *Infection, Genetics and Evolution*.

[5] Zhang L., Jie H., Xiao Y., Zhou C., Lyu W., Bai W. (2019). Genomic identification and expression analysis of the cathelicidin gene family of the forest musk deer. *Animals*.

[6] de la Fuente-Nunez C., Silva O. N., Lu T. K., Franco O. L. (2017). Antimicrobial peptides: role in human disease and potential as immunotherapies. *Pharmacology & Therapeutics*.

[7] Zhang G., Sunkara L. T. (2014). Avian antimicrobial host defense peptides: from biology to therapeutic applications. *Pharmaceuticals (Basel)*.

[8] Liu C., Jiang L., Liu L. (2018). Induction of avian *β*-defensin 2 is possibly mediated by the p38 MAPK signal pathway in chicken embryo fibroblasts after Newcastle disease virus infection. *Frontiers in Microbiology*.

[9] van Dijk A., Veldhuizen E. J., Haagsman H. P. (2008). Avian defensins. *Veterinary Immunology and Immunopathology*.

[10] van Dijk A., Veldhuizen E. J., Kalkhove S. I., Tjeerdsma-van Bokhoven J. L., Romijn R. A., Haagsman H. P. (2007). The *β*-defensin gallinacin-6 is expressed in the chicken digestive tract and has antimicrobial activity against food-borne pathogens. *Antimicrobial Agents and Chemotherapy*.

[11] Rodrigues I., Choct M. (2018). The foregut and its manipulation via feeding practices in the chicken. *Poultry Science*.

[12] Bailleul G., Guabiraba R., Virlogeux-Payant I. (2019). Systemic administration of avian defensin 7: distribution, cellular target, and antibacterial potential in mice. *Frontiers in Microbiology*.

[13] Elhamouly M., Nii T., Isobe N., Yoshimura Y. (2019). Age-related modulation of the isthmic and uterine mucosal innate immune defense system in laying hens. *Poultry Science*.

[14] Lynn D. J., Higgs R., Gaines S. (2004). Bioinformatic discovery and initial characterisation of nine novel antimicrobial peptide genes in the chicken. *Immunogenetics*.

[15] Xiao Y., Hughes A. L., Ando J. (2004). A genome-wide screen identifies a single beta-defensin gene cluster in the chicken: implications for the origin and evolution of mammalian defensins. *BMC Genomics*.

[16] Xiao Y., Cai Y., Bommineni Y. R. (2006). Identification and functional characterization of three chicken cathelicidins with potent antimicrobial activity. *Journal of Biological Chemistry*.

[17] Zou K. H., Tuncali K., Silverman S. G. (2003). Correlation and simple linear regression. *Radiology*.

[18] Pan D., Yu Z. (2014). Intestinal microbiome of poultry and its interaction with host and diet. *Gut Microbes*.

[19] Meade K. G., Higgs R., Lloyd A. T., Giles S., O’Farrelly C. (2009). Differential antimicrobial peptide gene expression patterns during early chicken embryological development. *Developmental & Comparative Immunology*.

[20] Terada T., Nii T., Isobe N., Yoshimura Y. (2018). Changes in the Expression of Avian *β*-defensins (AvBDs) and Proinflammatory Cytokines and Localization of AvBD2 in the Intestine of Broiler Embryos and Chicks during Growth. *Journal of Poultry Science*.

[21] Barshira E., Friedman A. (2006). Development and adaptations of innate immunity in the gastrointestinal tract of the newly hatched chick. *Developmental & Comparative Immunology*.

[22] Meade K. G., O'Farrelly C. (2019). *β*-Defensins: Farming the Microbiome for Homeostasis and Health. *Frontiers in Immunology*.

[23] Chairatana P., Nolan E. M. (2017). Defensins, lectins, mucins, and secretory immunoglobulin A: microbe-binding biomolecules that contribute to mucosal immunity in the human gut. *Critical Reviews in Biochemistry and Molecular Biology*.

[24] Ehmann D., Wendler J., Koeninger L. (2019). Paneth cell *α*-defensins HD-5 and HD-6 display differential degradation into active antimicrobial fragments. *Proceedings of National Academy of Sciences of the United States of America*.

[25] Jarczak J., Kosciuczuk E. M., Lisowski P. (2013). Defensins: natural component of human innate immunity. *Human Immunology*.

[26] Takakuwa A., Nakamura K., Kikuchi M. (2019). Butyric acid and leucine induce *α*-defensin secretion from small intestinal Paneth cells. *Nutrients*.

[27] Wang L., Li J., Li J. (2016). Identification of the Paneth cells in chicken small intestine. *Poultry Science*.

[28] Xiao Y., Hughes A. L., Ando J. (2004). A genome-wide screen identifies a single *β*-defensin gene cluster in the chicken: implications for the origin and evolution of mammalian defensins. *BMC Genomics*.

[29] Hong Y. H., Song W., Lee S. H., Lillehoj H. S. (2012). Differential gene expression profiles of *β*-defensins in the crop, intestine, and spleen using a necrotic enteritis model in 2 commercial broiler chicken lines. *Poultry Science*.

[30] Higgs R., Lynn D. J., Cahalane S. (2007). Modification of chicken avian *β*-defensin-8 at positively selected amino acid sites enhances specific antimicrobial activity. *Immunogenetics*.

[31] Robinson K., Deng Z., Hou Y., Zhang G. (2015). Regulation of the intestinal barrier function by host defense peptides. *Frontiers in Veterinary Science*.

[32] Mowbray C. A., Niranji S. S., Cadwell K., Bailey R., Watson K. A., Hall J. (2018). Gene expression of AvBD6-10 in broiler chickens is independent of AvBD6, 9, and 10 peptide potency. *Veterinary Immunology and Immunopathology*.

[33] Sunkara L. T., Achanta M., Schreiber N. B. (2011). Butyrate enhances disease resistance of chickens by inducing antimicrobial host defense peptide gene expression. *PLoS One*.

