# Supplementary material for: Developmental and Tissue Patterns of the Basal Expression of Chicken Avian β-Defensins
Source: Biomed Res Int. 2020 Dec 14;2020:2567861. doi: 10.1155/2020/2567861 (PMC7787727; doi:10.1155/2020/2567861)
Supplement: Supplementary Materials — Supplementary Figure: basal expression of thirteen chicken AvBDs in the GI tract, liver, and spleen. Tissue segments were collected from 7-day-old chickens. After RNA extraction and reverse transcription, AvBD expression levels were determined by real-time PCR using gene-specific primers. The expression levels of all AvBDs were calculated relative to that of the colon using GAPDH as the reference gene and expressed as the mean of nine chickens for each time point. [file 2567861.f1.docx]

**Supplementary Figure. Basal expression of thirteen chicken *AvBD*s in the GI tract, liver and spleen.** Tissue segments were collected from 7-day-old chickens. After RNA extraction and reverse transcription, *AvBD*s expression levels were determined by real-time PCR using gene-specific primers. The expression levels of all *AvBD*s were calculated relative to that of the colon using *GAPDH* as reference gene and expressed as mean of nine chickens for each time point.
